# Fellow-Workers and Their Doings

**Published:** 1914-05-23

**Authors:** 


### ROUND THE WORLD.

### Fellow-Workers and their Doings.

**[The Editor Invites Early Information and Contributions Marked "Fellow-Workers."]**

Many fellow-workers owing allegiance to Edinburgh University met at the University Club dinner held under the presidency of Lord Balfour of Burleigh at the Hôtel Cecil last week. Of the distinguished medical practitioners present on that occasion, Sir William Turner, K.C.B., received a special ovation. The venerated principal of Edinburgh University, who in his early days was recognised as one of the greatest scientific teachers Scotland ever produced, maintains his hold not only on the intellects, but on the affections of all Edinburgh men to a remarkable extent. Qualifying M.R.C.S. from "Bart.'s" as long ago as 1853, Sir William Turner particularly distinguished himself as an anatomist, and his lectures on this subject at Edinburgh never failed to attract a large following. Few men can have exceeded in number the honorary academic distinctions that have been bestowed on him.

Everyone is glad to know that Dr. W. Ainslie Hollis, of Hove, has done so well after his recent operation for appendicitis. By accepting the presidency of the British Medical Association last year Dr. Hollis incurred great responsibilities towards the members of his profession and much extra work. His presidential address at the Brighton Meeting (1913) on the medicinal history of that town was full of much interest and widely read. Dr. Hollis spent his student days at Cambridge and at St. Bartholomew's Hospital, taking the M.D. of his University in 1871 and being elected F.R.C.P. Lond. five years later; he was for many years a member of the active staff of the Sussex County Hospital, to which institution he is consulting physician.

Mr. Hugh Lett, M.B., F.R.C.S., has been investigating the relation between threadworms and appendicitis. In an interesting *résumé* of statistics published in the *Practitioner* (May 1914) he mentions that, out of a thousand cases of appendicitis operated on at the London Hospital recently, threadworms were found only five times. He notes the disparity of his observations as compared with those of various foreign investigators, one of whom, Rheindorf, has stated that the parasites in question occur in 47 per cent. of appendical inflammation in children. Mr. Lett, who is surgeon to out-patients at the "London," points out that careful statistics should distinguish between appendicitis in young people and in adults, because threadworms are a particular ailment of early years, and a wrong impression may be given by mixing the cases indiscriminately for purposes of report. In support of this contention it is noted that, in all the cases of appendicitis associated with threadworms in his series at the London Hospital, the patients were under fifteen years of age.

Dr. H. C. Corry Mann, M.D., M.R.C.P., of "Guy's," has been carrying out important observations on infant feeding during the past three or four years, having given particular attention to the dietetic treatment of rickets. As physician to the out-patient department at the Evelina Hospital for Sick Children, Dr. Mann has been able to note the effects of different kinds of feeding in many hundreds of rachitic children. In the *Guy's Hospital Gazette* this investigator reports in favour of feeding infants with undiluted citrate milk, citing many interesting cases in support of his theory that the best treatment for rickets is the administration of fresh cows' milk, to which sodium citrate and malt have been added.

Professor J. Rutherford Morison's withdrawal from the active staff of the Royal Victoria Infirmary at Newcastle is greatly regretted by the students of Durham University. The retiring surgeon, who is Professor of Surgery of the Durham University, is well known as a clinical teacher of wide experience, and in the course of his arduous work has found time to write much that has been of interest to practitioners. Professor Morison studied medicine at Birmingham, Edinburgh, and Vienna. Qualifying L.R.C.S. Edin. in 1874, he subsequently became F.R.C.S. in 1879 and F.R.C.S. Eng. in 1890.

The new anæsthetist to the West London Hospital, Hammersmith, Dr. W. E. Robinson, has been working for some time at that institution as assistant anæsthetist. An old "Guy's" man, Dr. Robinson, who is an M.D. Oxon., qualified by taking the diploma of the Conjoint Board in 1894; he is also a visiting anæsthetist at the Royal Waterloo Hospital for Children and Women, and has done work at the Infants' Hospital, Westminster.

Considerable credit is due to Dr. A. Helen Boyle for her original work in endeavouring to better present-day methods of treating early mental cases. Dr. Helen Boyle qualified from the London School of Medicine for Women by taking the triple Edinburgh and Glasgow diploma in 1893, and subsequently became M.D. Brux. with distinctions. For some years she has given special attention to the early treatment of mental and nervous cases, and has lately been instrumental in founding a mental hospital at Brighton. As assistant medical officer to Claybury Asylum, Dr. Boyle had considerable experience of her special subject.

Dr. Jamieson Boyd Hurry has further associated himself with the public life of Reading through the new gymnasium which he and his wife have presented to the University College there. Dr. Hurry, who has retired from active practice, is perhaps best known to the profession at large from his interesting studies of vicious circles in disease. Reading medicine at Cambridge and "Bart.'s" Dr. Hurry qualified in 1882, and is an M.D. Cantab., D.P.H., and M.R.C.S. Eng. Sometime surgeon to the Reading Dispensary, he was also medical officer to Reading University College, and is a J.P. for the district.

A subject of widespread interest, neurasthenia, has been chosen by Dr. Theodore Stacey Wilson for the Ingleby Lectures of Birmingham University. The Ingleby Lecturer for this year is a well-known Birmingham physician on the staff of the General Hospital there; he is a gold medallist of Edinburgh, where he took his M.D. in 1892, as well as an F.R.C.P. Lond.

The Salford Children's and Northern Hospitals at Manchester have elected Dr. A. H. Miller, M.D. Cantab., M.R.C.P. Lond., to be their joint pathologist. Trained at Guy's Hospital, Dr. Miller subsequently studied in Berlin, and was at one time physician to out-patients at the City of London Hospital for Diseases of the Chest at Victoria Park.

